# The impact of primary percutaneous coronary intervention timing on prognosis in STEMI patients

**DOI:** 10.1016/j.clinme.2026.100602

**Published:** 2026-06-05

**Authors:** Lihui Zhou, Jin Si, Ming Yi, Keling Xiao, Lijie Sun, Haoyu Zhang, Jinghao Sun, Zhenxing Fan, Zhi Liu, Jing Gao, Lin Pi, Yinghua Zhang, Jing Li

**Affiliations:** a Department of Geriatrics, Xuanwu Hospital Capital Medical University, National Clinical Research Center for Geriatric Disease, Beijing, China; b Department of Cardiology, Beijing Chaoyang Hospital, Capital Medical University, China; c Beijing Municipal Geriatric Medical Research Center, Beijing, China; d Department of Emergency, Xuanwu Hospital Capital Medical University, Beijing, China; e Department of Cardiology, Xuanwu Hospital Capital Medical University, Beijing, China; f Department of Cardiology, Chuiyangliu Hospital Affiliated with Tsinghua University, Beijing, China

**Keywords:** Primary percutaneous coronary intervention, ST-segment elevation myocardial infarction, Timing, Long-term outcome

## Abstract

**Background:**

The benefit of primary percutaneous coronary intervention (PPCI) performed beyond 6 h after ST-segment elevation myocardial infarction (STEMI) symptom onset remains uncertain.

**Methods:**

Patients presenting with STEMI from January 2009 to December 2022 were consecutively enrolled and stratified into three groups: PPCI within 6 h, PPCI beyond 6 h, and no PPCI. Clinical outcomes were compared using Cox multivariable analyses and propensity score matching.

**Results:**

A total of 3,720 patients were included (77.4% male, mean age 62.61 ± 12.66 years). Patients were divided into PPCI within 6 h group (n = 1,127, 30.3%), PPCI beyond 6 h group (n = 587, 15.8%), and no PPCI group (n = 2,006, 53.9%). In-hospital mortality rates were 2.8%, 4.1% and 5.2%, respectively (p = 0.006), with pairwise analysis revealing significant differences among the groups. During a median follow-up of 4.96 years, cumulative mortality was 13.1% in PPCI within 6 h group, 16.0% in PPCI beyond 6 h group, and 24.0% in no PPCI group (P < 0.001). PPCI within 6 h was associated with lower mortality (adjusted hazard ratio (HR) 0.565 (0.429–0.744), p < 0.001) or MACCE (adjusted HR 0.765 (0.640–0.914), p = 0.003) while PPCI beyond 6 h showed comparable cumulative mortality (adjusted HR 0.766 (0.575–1.020), p = 0.068) and MACCE (adjusted HR 0.884 (0.721–1.083), p = 0.234) versus no PPCI. Concordant results were observed in propensity score-matched cohorts.

**Conclusion:**

While PPCI beyond 6 h of symptom onset may improve in-hospital outcomes in STEMI patients, it is necessary to perform PPCI within 6 h to achieve significant long-term benefits.

## Highlights

• Early PPCI (≤6 h) reduces infarct size and improves survival, underscoring its critical time-dependency.
• PPCI at 6–12 h still offers short-term benefit by restoring coronary blood flow and limiting ischaemia.
• Long-term outcomes decline with delay, highlighting that timely reperfusion remains key for STEMI.
• Prompt PPCI initiation post-symptom onset optimises both immediate and long-term patient results.

## Graphical abstract

**Figure:**
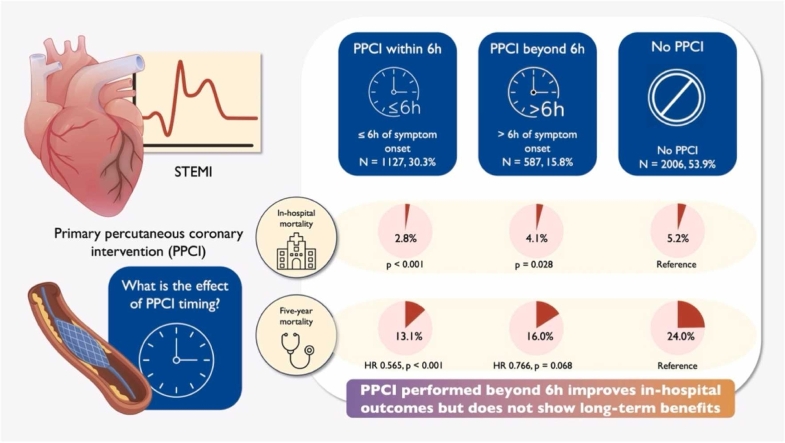
Although PPCI within 6 h of symptom onset is strongly associated with reduced infarct size and improved survival, PPCI beyond 6 h may still confer short-term in-hospital benefits by restoring coronary perfusion and mitigating ongoing ischaemia. However, its impact on long-term outcomes is significantly attenuated. The graphical abstract was prepared using Adobe Illustrator and Microsoft PowerPoint. Abbreviations: HR, hazard ratio; PPCI, primary percutaneous coronary intervention; STEMI, ST-segment elevation myocardial infarction.

## Introduction

ST-segment elevation myocardial infarction (STEMI) remains a significant contributor to global cardiovascular morbidity and mortality.^1^ Early reperfusion of the occluded infarct-related artery (IRA) is the most effective way to minimise myocardial injury, preserve ventricular function and reduce long-term complications.^2^ The currently guideline-recommended ʻ12 h’ time window is based on a systematic analysis of cumulative evidence from over a dozen randomised controlled trials of fibrinolytic therapy conducted between the 1980s and 1990s.^3^, ^4^ Subsequently, this time frame was adapted for use in PPCI. Currently, international guidelines prioritise PPCI as the preferred reperfusion strategy for STEMI, with thrombolytic therapy as an alternative when PPCI is not promptly feasible.^5^ It remains uncertain whether the time window established for thrombolytic therapy is directly applicable to PPCI.

Experimental studies have demonstrated wavefront myocyte necrosis following coronary ligation, with transmural necrosis observed at 38% after 40 min, 57% at 3 h, 71% at 6 h and 85% at 24 h, indicating that ischaemic myocardium is unlikely to be salvaged by reperfusion after 6 h of coronary occlusion.^6^ Besides, prolonged ischaemia is a critical determinant of myocardial ischaemia-reperfusion injury, which is characterised by the paradoxical worsening of myocardial damage despite the restoration of blood flow.^7^ Clinical studies have further shown that a total ischaemic time greater than 6 h independently predicts the no-reflow phenomenon.^8^, ^9^ Large-scale studies have demonstrated that reperfusion therapy achieves optimal efficacy within 4 h of symptom onset,^10^, ^11^ while when the time interval from symptom onset to PPCI exceeds 6 h, there is a significant increase in the combined endpoint of all-cause mortality and hospitalisation for heart failure among STEMI patients.^12^ Both experimental models and large-scale clinical data consistently reveal that reperfusion after ischaemia lasting 6–12 h falls within the ʻhigh-damage window’, a period associated with heightened myocardial injury.^13^, ^14^ Even within the recommended time windows outlined in clinical guidelines, the effectiveness of reperfusion therapy may vary when performed within or beyond the 6-h threshold.

The purpose of the present study was to compare in-hospital and long-term outcomes in STEMI patients receiving PPCI within 6 h, beyond 6 h and those not receiving PPCI, exploring the impact of reperfusion strategy and PPCI timing on STEMI prognosis, and offering insights into improving STEMI care.

## Methods

### Study population

Patients hospitalised for STEMI were retrospectively included in a single centre from January 2009 to December 2022. STEMI was defined as ischaemic symptoms lasting ≥20 min, accompanied by electrocardiographic evidence of significant ST-segment elevation or STEMI-equivalent findings, and elevated myocardial biomarkers above the 99th percentile upper reference limit. Significant ST-segment elevation was defined as new ST-segment elevation at the J point in at least two contiguous leads, with thresholds of ≥2 mm in precordial leads or ≥1 mm in limb leads. The exclusion criteria were as follows: 1) previous myocardial infarction; 2) receiving fibrinolytic therapy; 3) receiving coronary artery bypass graft; 4) refusal of medical records used for research purposes. Patients were categorised into three groups according to reperfusion strategy and timing: PPCI within 6 h, PPCI beyond 6 h, and no PPCI. PPCI was defined as emergent primary PCI during the acute phase. Patients were classified based on whether and when PPCI was performed after symptom onset; those not receiving emergent PPCI were assigned to the no PPCI group, irrespective of subsequent elective revascularisation. All patients received optimal medical therapy following contemporary guidelines. Patients’ information (clinical characteristics, calendar year of the index hospitalisation, laboratory results and angiography) was collected through enquiry and review of medical records.

### Biochemical measurements

Peripheral blood samples were obtained within 24 h of hospital admission. Serum levels of glycosylated haemoglobin (HbA1c), low-density lipoprotein cholesterol (LDL-C), hypersensitive C-reactive protein (hs-CRP) and interleukin-6 (IL-6) were measured using standardised laboratory protocols. Cardiac troponin I (cTnI) levels were monitored every 6 hours during the first 24 h, with the highest recorded values used for analysis.

### Revascularisation

A loading dose of aspirin and P2Y12 receptor inhibitors was administered, followed by diagnostic coronary angiography through either a radial or femoral approach in eligible patients by certified interventionists. Anatomical and functional assessments of the coronary arteries were performed to identify the IRA and determine the revascularisation strategy. Single-vessel disease was defined as stenosis of at least 50% in one major epicardial coronary artery, while multi-vessel disease was defined as stenosis of at least 50% in ≥ two major epicardial coronary arteries. Stent implantation of IRA was at the discretion of the operator, depending on the lesions’ features, with optimal angioplasty defined as residual stenosis <10%. Non-infarct-related artery lesions were treated electively. In cases of no-reflow, pharmacological interventions, including vasodilators such as sodium nitroprusside or diltiazem, were administered to improve coronary perfusion.

### Endpoint and follow-up

All patients were followed up by contacts through telephone calls, outpatient visits, and review of the electronic medical records for clinical characteristics every 6 months.

In-hospital mortality was defined as all-cause death during the index hospitalisation. Long-term endpoints included all-cause mortality and major adverse cardiovascular and cerebrovascular events (MACCE). MACCE was defined as a composite of cardiac death, non-fatal myocardial infarction, unplanned repeat revascularisation, heart failure hospitalisation and stroke. Planned staged or elective PCI performed as part of the initial treatment strategy was not counted as a MACCE event.

### Statistical analysis

Continuous variables were expressed as mean ± SD if normally distributed and median and interquartile range (IQR) if abnormally distributed. Categorical variables were expressed as frequencies with percentages. As appropriate, the Fisher’s exact test, Student’s *t*-test, Mann–Whitney test, and Kruskal–Wallis test were used to compare categorical and continuous variables.

Survival curves were generated using the Kaplan–Meier method and compared using log-rank tests. To evaluate the independent relationship between reperfusion therapy type and outcomes, a fully adjusted Cox regression analysis was conducted, including age, sex, calendar year of index hospitalisation, hypertension, diabetes, current smoking, obesity, Killip class ≥2, multivessel disease, left ventricular ejection fraction (LVEF), IL-6 and hsCRP as covariates to account for potential confounders.

To reduce confounding due to baseline differences between groups, propensity score matching was performed separately for PPCI beyond 6 h versus PPCI within 6 h and PPCI beyond 6 h versus no PPCI comparison. Propensity scores were estimated using logistic regression models with group assignment as the dependent variable. The covariates included in each propensity score model were age, sex, hypertension, diabetes, current smoking, obesity, Killip class ≧2, multivessel disease, GRACE score, LVEF, IL-6 and calendar year of index hospitalisation. Missing baseline covariate values were imputed using mean imputation before propensity score estimation. A 1:1 nearest-neighbour matching algorithm was applied without replacement, using a caliper equal to 0.5 standard deviations of the estimated propensity score. Covariate balance before and after matching was assessed using standardised mean differences. An absolute standardised mean difference of less than 0.10 was considered to indicate acceptable balance. Time-to-event outcomes were analysed in the propensity score-matched cohorts using Cox proportional hazards models with matching weights and robust variance estimation clustered by matched subclass.

Statistical analyses were performed with SPSS 29 (IBM SPSS Statistics 29, IBM Corporation, Armonk, NY, USA) and R 4.6.0 (Posit Software, Boston, USA). All tests were two-sided, and differences were considered statistically significant at p-values <0.05.

## Results

### Study population and baseline characteristics

A total of 3,720 consecutive STEMI patients were enrolled in this study, with 2,879 men (77.4%) and a mean age of 62.61 ± 12.66 years. Patients were divided into three groups based on the strategy and timing of reperfusion: the PPCI within 6 h group (n = 1,127), the PPCI beyond 6 h group (n = 587), and the no PPCI group (n = 2,006). Delayed PPCI or absence of PPCI was attributable to both system-related and patient-related factors, including late presentation, delayed transfer, haemodynamic instability, patient refusal and physician judgement.

Baseline demographic, clinical, laboratory, angiographic and procedural characteristics are shown in Table 1.

**Table 1:** Baseline characteristics and early management according to reperfusion strategy and timing of primary percutaneous coronary intervention.

Variables	Group A (n = 1,127)	Group B (n = 587)	Group C (n = 2,006)	P value
Demographics				
Age, year	60.62±12.32	62.14±12.15	63.86±12.84	**<0.001**
Male, (n, %)	920 (81.6)	454 (77.3)	1,505 (75.0)	**<0.001**
Calendar year of index hospitalisation	2,017 (2,014, 2,020)	2,018 (2,015, 2,021)	2,016 (2,014, 2,019)	**<0.001**
Cardiovascular risk factors				
Hypertension, n (%)	590 (52.4)	325 (55.4)	1,208 (60.2)	**<0.001**
Diabetes mellitus, n (%)	289 (25.6)	199 (34.0)	670 (33.4)	**<0.001**
Current smoking, n (%)	702 (62.3)	343 (58.4)	1,213 (60.5)	0.286
Obesity, n (%)	259 (23.0)	111 (18.9)	419 (20.9)	0.173
Clinical presentation				
Anterior infarction, n (%)	587 (52.1)	313 (53.3)	1,074 (53.5)	0.729
GRACE risk score	145.74±35.47	149.72±35.86	151.36±37.59	**0.001**
Killip class ≧2, n (%)	422 (37.4)	228 (38.8)	874 (43.6)	**0.002**
Cardiogenic shock	83 (7.4)	46 (7.8)	173 (8.6)	**0.004**
Early management				
Total ischaemic time, h	3.98 (2.97, 4.98)	8.50 (7.00, 12.43)	-	**<0.001**
S-FMC, h	3.00 (2.00, 4.00)	7.00 (6.00, 11.00)	11.00 (4.00, 24.00)	**<0.001**
D-to-B, min	60.00 (40.00, 74.00)	60.00 (45.00, 103.00)	-	**<0.001**
Aspirin, n (%)	1,125 (99.8)	582 (99.1)	1,978 (98.6)	**0.005**
P2Y12 inhibitor, n (%)	1,107 (98.2)	581 (98.9)	1,982 (98.8)	0.080
Glycoprotein IIb/IIIa inhibitor, n (%)	526 (46.7)	271 (46.2)	176 (8.8)	**< 0.001**
Bivalirudin, n (%)	11 (1.0)	9 (1.5)	72 (3.6)	**<0.001**
Statin, n (%)	1,116 (99.0)	579 (98.7)	1,966 (98.0)	0.151
ACEI/ARB/ARNI, n (%)	992 (88.0)	514 (87.6)	1,803 (89.9)	0.179
Beta-blocker, n (%)	888 (78.8)	435 (74.1)	1,567 (78.1)	0.074
Coronary angiography and reperfusion				
Multi-vessel disease, n (%)	717 (63.6)	414 (70.5)	1,466 (73.1)	**< 0.001**
IRA, n (%)				**<0.001**
LAD	539 (47.8)	286 (48.7)	1,001 (49.9)	
LCX	132 (11.7)	89 (15.2)	353 (17.6)	
RCA	456 (40.5)	212 (36.1)	652 (32.5)	
Emergent stent implantation	1.11±0.60	1.14±0.65	-	0.413
Post-PPCI TIMI flow = 3	1,075 (95.4)	545 (92.9)	-	**0.004**
Elective PCI, n (%)	211 (18.7)	117 (19.9)	1,393 (69.4)	**<0.001**
Auxiliary examination				
LVEF, %	55.11±9.47	54.06±9.48	53.09±10.65	**<0.001**
cTnI, ng/mL	34.75 (16.68, 50.00)	32.20 (12.00, 50.00)	7.32 (2.04, 17.85)	**<0.001**
NT-proBNP, pg/mL	109 (38, 299)	468 (156, 1,334)	793 (181, 3,117)	**<0.001**
LDL-C, mmol/L	2.85±0.86	2.81±0.91	2.66±0.88	**<0.001**
HbA1c, %	6.50±1.93	6.84±2.78	6.68±1.97	**0.007**
hs-CRP, mg/L	3.76 (1.60, 8.65)	6.63 (2.15, 15.37)	7.73 (2.36, 19.55)	**<0.001**
IL-6, pg/mL	16.43 (8.70, 30.15)	21.97 (10.56, 38.7)	22.40 (10.11, 48.64)	**<0.001**

ACEI, angiotensin-converting enzyme inhibitor; AMI, acute myocardial infarction; ARB, angiotensin receptor blocker; ARNI, angiotensin receptor-neprilysin inhibitor; cTnI, cardiac troponin I; GRACE score, Global Registry of Acute Coronary Events score; HbA1c, glycosylated haemoglobin; hs-CRP, high sensitive C-reactive protein; IL-6, interleukin-6; IRA, infarct-related artery; LAD, left anterior descending; LCX, left circumflex; LDL-C, low-density lipoprotein cholesterol; LVEF, left ventricular ejection fraction; NT-proBNP, N-terminal pro-brain natriuretic peptide; PPCI, primary percutaneous coronary artery intervention; RCA, right coronary artery.

Patients in the PPCI beyond 6 h group and the no PPCI group were generally older and exhibited a higher incidence of cardiogenic shock, greater burden of cardiovascular risk factors and comorbidities, a higher prevalence of multi-vessel coronary artery disease, reduced LVEF, and elevated levels of inflammatory markers compared to those who underwent PPCI within 6 h of symptom onset. P2Y12 inhibitors, statins, renin-angiotensin-aldosterone system inhibitors and beta blockers were administered in similar proportions across all three groups. Aspirin was more frequently used in patients undergoing PPCI within 6 h. A higher proportion of patients in the early and PPCI beyond 6 h groups received glycoprotein IIb/IIIa inhibitors, whereas bivalirudin was more commonly administered in the no PPCI group. Compared with patients receiving PPCI within 6 h, those without PPCI were treated in earlier calendar years, whereas those receiving PPCI beyond 6 h were treated in later calendar years.

### In-hospital outcome

Among the total study cohort, there were 159 deaths (4.3%) during hospitalisation, with 31 deaths (2.8%) in the PPCI within 6 h group, 24 deaths (4.1%) in PPCI beyond 6 h group, and 104 deaths (5.3%) in no PPCI group (p = 0.006). Pairwise comparisons among the groups demonstrated statistically significant differences: PPCI within 6 h versus no PPCI (p < 0.001) and PPCI beyond 6 h versus no PPCI (p = 0.028).

### Long-term outcome

The median follow-up duration is 4.96 years, with a follow-up rate of 91.71%. Baseline characteristics of patients lost to follow-up and those retained in the analysis were compared and are presented in Supplementary Table 1. Patients lost to follow-up had an earlier calendar year of index hospitalisation and a lower prevalence of hypertension; no other systematic differences were observed between the two groups.

**Fig. 1:**
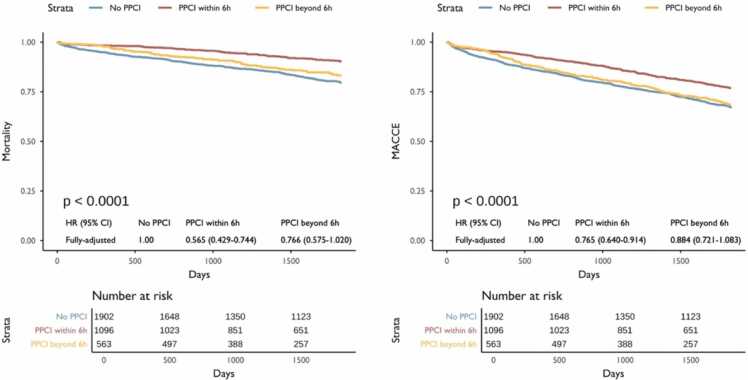
Five-year survival according to type of reperfusion strategy and timing of primary percutaneous coronary intervention. Abbreviations: CI, confidence interval; HR, hazard ratio; PPCI, primary percutaneous coronary intervention.

The estimated 5‐year survival rates were 90.3% (95% CI, 88.3–92.3%) in the PPCI within 6 h group, 86.6% (95% CI, 83.2–90.0%) in the PPCI beyond 6 h group, and 79.5% (95% CI, 77.5–81.5%) in the no PPCI group (p < 0.001). Corresponding 5‐year MACCE-free survival rates were 76.8% (95% CI, 74.1–79.6%), 71.6% (95% CI, 67.5–76.0%) and 67.0% (95% CI, 64.8–69.3%), respectively (p < 0.001). The Kaplan–Meier curves for mortality and MACCE are depicted in Fig. 1.

Multivariable-adjusted analyses are summarised in Table 2, Table 3. PPCI within 6 h was associated with significantly lower risks of all-cause mortality (adjusted HR, 0.565; 95% CI, 0.429–0.744; p < 0.001) and MACCE (adjusted HR, 0.765; 95% CI, 0.640–0.914; p = 0.003). In contrast, PPCI beyond 6 h was not significantly associated with reduced risks of all-cause mortality (adjusted HR, 0.766; 95% CI, 0.575–1.020; p = 0.068) or MACCE (adjusted HR, 0.884; 95% CI, 0.721–1.083; p = 0.234) compared with no PPCI.

**Table 2:** Multivariate Cox regression of long-term mortality.

Variates	HR	95% CI	P value
Reperfusion strategy			**<0.001**
PPCI within 6 h	0.565	0.429–0.744	<0.001
PPCI beyond 6 h	0.766	0.575–1.020	0.068
Age	1.070	1.060–1.081	<0.001
Sex	1.043	0.812–1.341	0.740
Calendar year of index hospitalisation	0.993	0.950–1.037	0.736
Hypertension	1.048	0.850–1.293	0.659
Diabetes mellitus	1.565	1.279–1.917	<0.001
Current smoking	1.194	0.940–1.518	0.147
Obesity	1.072	0.824–1.394	0.607
Killip class ≧2	1.399	1.121–1.746	0.003
Multivessel disease	0.899	0.661–1.223	0.498
LVEF	0.958	0.948–0.968	<0.001
hsCRP	1.015	1.008–1.022	<0.001
IL-6	1.000	1.000–1.001	0.436

The no PPCI group was used as the reference group for reperfusion strategy.

CI, confidence interval; HR, hazard ratio; hsCRP, high-sensitivity C-reactive protein; IL-6, interleukin-6; LVEF, left ventricular ejection fraction; PPCI, primary percutaneous coronary intervention.

**Table 3:** Multivariate Cox regression of long-term MACCE.

Variates	HR	95% CI	P value
Reperfusion strategy			**0.012**
PPCI within 6 h	0.765	0.640–0.914	0.003
PPCI beyond 6 h	0.884	0.721–1.083	0.234
Age	1.034	1.027–1.041	<0.001
Sex	0.961	0.790–1.169	0.689
Calendar year of index hospitalisation	1.026	0.995–1.058	0.100
Hypertension	1.170	1.006–1.361	0.042
Diabetes mellitus	1.264	1.088–1.467	0.002
Current smoking	1.100	0.923–1.312	0.288
Obesity	1.161	0.970–1.391	0.103
Killip class ≧2	1.158	0.992–1.351	0.062
Multivessel disease	1.127	0.922–1.377	0.243
LVEF	0.975	0.968–0.983	<0.001
hsCRP	1.010	1.005–1.015	<0.001
IL-6	1.000	1.000–1.001	0.154

The no PPCI group was used as the reference group for reperfusion strategy.

CI, confidence interval; HR, hazard ratio; hsCRP, high-sensitivity C-reactive protein; IL-6, interleukin-6; LVEF, left ventricular ejection fraction; PPCI, primary percutaneous coronary intervention.

The propensity score-matched cohorts were well balanced, with covariate balance diagnostics presented in Supplementary Tables 2–5. A total of 572 matched pairs were retained for the comparison between PPCI within 6 h and PPCI beyond 6 h, and 565 matched pairs were retained for the comparison between PPCI beyond 6 h and no PPCI. Time-to-event outcomes were then analysed in the matched cohorts.

**Fig. 2:**
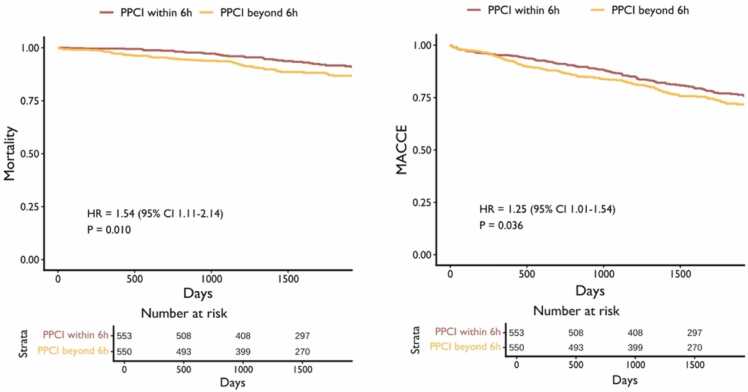
Five-year survival in propensity score-matched PPCI within 6 h and PPCI beyond 6 h populations. Abbreviations: PPCI, primary percutaneous coronary intervention.

Among patients included in the comparison between PPCI within 6 h and PPCI beyond 6 h, 142 deaths and 342 MACCE events occurred during post-discharge follow-up. Compared with PPCI within 6 h, PPCI beyond 6 h was associated with a significantly higher risk of post-discharge death (HR, 1.54; 95% CI, 1.11–2.15; p = 0.010) and MACCE (HR, 1.25; 95% CI, 1.02–1.54; p = 0.036) (Fig. 2).

**Fig. 3:**
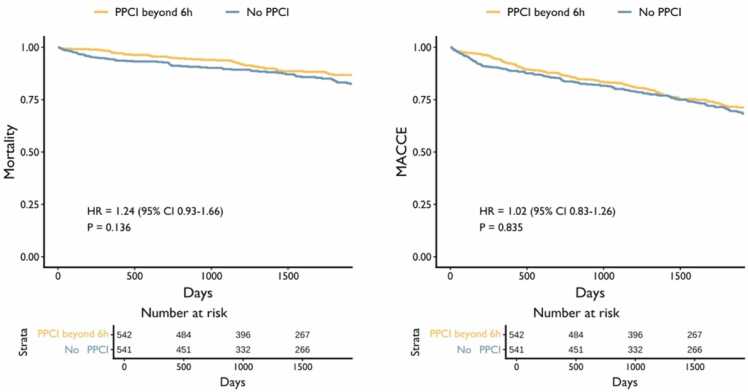
Five-year survival in propensity score-matched PPCI beyond 6 h and no PPCI populations. Abbreviations: PPCI, primary percutaneous coronary intervention.

In the matched comparison between PPCI beyond 6 h and no PPCI, 182 deaths and 368 MACCE events were recorded during post-discharge follow-up. No PPCI was not associated with a significantly increased risk of post-discharge death compared with PPCI beyond 6 h (HR, 1.24; 95% CI, 0.93–1.66; p = 0.136). Similarly, the risk of post-discharge MACCE did not differ significantly between the two groups (HR, 1.02; 95% CI, 0.83–1.26; p = 0.835) (Fig. 3).

## Discussion

The present study investigated the effects of PPCI timing on the prognosis of STEMI patients, and the key findings are as follows: 1) approximately one-third of the STEMI patients received PPCI within 6 h from symptom onset; 2) PPCI, whether performed within or beyond 6 h, was associated with more favourable in-hospital outcomes compared with no PPCI; 3) PPCI administered beyond 6 h fails to confer long-term prognostic benefits.

STEMI is precipitated by sudden coronary occlusion, leading to myocardial ischaemia and a cellular energy crisis, with sustained ischaemia beyond 20–30 min inducing irreversible cardiomyocyte necrosis.^15^ Despite substantial therapeutic progress, current clinical practice still falls short of optimal evidence-based standards, and achieving early reperfusion remains a significant challenge. Data from the China PEACE-Retrospective Acute Myocardial Infarction Study showed that the national reperfusion rates for STEMI were 55.2%, 53.9% and 55.0% in 2001, 2006 and 2011, respectively, with PPCI rates of 10.2%, 17.0%, and 27.6% during the same years.^16^ Such delays are influenced by both system-level factors – including transfer efficiency and procedural resource availability – and patient-related variables such as advanced age, time of symptom onset and geographic barriers, all of which critically affect the timing and outcomes of PPCI.^17^

Animal experiments revealed that, after complete coronary occlusion, approximately 71% myocardial necrosis was observed at 6 h, and further extension by 24 h, suggesting that the resuscitation of necrotic myocardium following reperfusion is highly limited with prolonged ischaemic duration. However, it remains uncertain whether the results from these preliminary studies can be effectively translated into clinical practice to confirm that reperfusion within 6 h is the most effective therapeutic strategy, due to the lack of comparable clinical investigations.

The results of the current study suggest that PPCI performed beyond 6 h after symptom onset was associated with more favourable in-hospital outcomes compared with no PPCI, although this association appeared less pronounced than that observed for PPCI performed within 6 h.

The primary mechanism is that reperfusion, even at later stages, plays a critical role in mitigating microvascular injury, attenuating the inflammatory response, facilitating metabolic recovery and minimising myocardial stunning.^18^, ^19^ In addition, Manfrini *et al* reported that PCI improved myocardial blood flow, including in remote regions, suggesting a potential physiological mechanism by which reperfusion may contribute beyond the directly treated lesion.^20^ Thus, reperfusion beyond 6 h remains a valuable intervention for improving short-term clinical outcomes, emphasising the importance of prompt coronary reperfusion in STEMI patients. The PRAGUE-2 trial demonstrated that primary PCI performed within 12 h significantly reduced 30-day mortality compared with on-site thrombolysis, suggesting that interventions administered between 6 and 12 h after symptom onset can still provide substantial clinical benefit, which is consistent with the main findings of the present study.^21^ Meanwhile, Topol *et al* assessed the effectiveness of late reperfusion therapy in patients with acute coronary occlusion 6–24 h after symptom onset. Coronary angioplasty was associated with an initial 81% recanalisation success and improved ventricular function at 1 month, but by late follow-up, no advantage could be demonstrated for this procedure.^22^

Several underlying factors may account for the prognostic differences observed between patients undergoing PPCI within 6 h and those treated beyond 6 h after symptom onset. The therapeutic efficacy of PPCI is principally attributed to its capacity to promptly restore blood flow in the occluded coronary artery, thereby attenuating myocardial ischaemia and preserving jeopardised myocardium. The preservation of myocardial tissue is critically time-dependent. In clinical research, a nearly linear relationship was observed between treatment delay and mortality. For every 10-min delay, an additional 0.34 deaths occurred per 100 haemodynamically stable patients treated with PPCI, and as much as 3.31 additional deaths occurred in patients with cardiogenic shock.^23^ Moreover, reperfusion of the ischaemic myocardium can paradoxically lead to reperfusion injury, and prolonged ischaemia exacerbates reperfusion damage.^7^, ^24^ The restoration of blood flow triggers leucocyte infiltration and pro-inflammatory cascades, which, while aiding tissue repair, can also impair healing and post-MI remodelling.^25^ Furthermore, even after successful revascularisation, cardiac tissue may still fail to achieve normal perfusion, a condition known as the ʻno-reflow phenomenon’, which is associated with a higher risk of cardiogenic shock, recurrent MI and increased mortality.^26^ No-reflow occurs predominantly when ischaemia persists for ≥90 min.^27^ In this study, the incidence of TIMI grade less than 3 after PPCI was 4.6% in the PPCI within 6 h group and 7.1% in the PPCI beyond 6 h group, the differences in prognosis between groups could be related to the degree of blood flow recovery after the procedure.

Surprisingly, findings from the present study indicate that PPCI performed beyond 6 h of symptom onset failed to confer a long-term prognostic advantage.

Although PPCI can temporarily stabilise patients’ vital signs following successful intervention, the delayed restoration of coronary perfusion is insufficient to prevent irreversible myocardial injury, thereby limiting its impact on long-term outcomes. The observed differences in LVEF and the incidence of CS among the three groups may partially account for the variation in mortality rates, as both reduced LVEF and the presence of CS are well-established predictors of adverse clinical outcomes in STEMI patients. Notably, it should be noted that 70% of patients in the no-PPCI group eventually received elective PCI, suggesting a delayed but planned revascularisation strategy rather than purely conservative management in a substantial proportion of cases. Given the limited myocardial salvage beyond 6 h of PPCI, long-term benefit in STEMI is likely driven by revascularisation of non-infarct-related arteries. The COMPLETE trial demonstrated that regardless of the timing of non-culprit-lesion PCI, complete revascularisation was superior in reducing cardiovascular death or myocardial infarction.^28^ Similarly, the CvLPRIT trial found that index admission complete revascularisation significantly lowered the rate of the composite primary endpoint at 12-month follow-up, possibly due to preventing hibernation and improving myocardial salvage and attenuation of early myocardial stunning by enhancing blood flow to watershed areas of infarction.^29^ Finally, greater emphasis is placed on secondary prevention, with a focus on long-term guideline-directed secondary prevention medications and lifestyle modifications to improve patient prognosis. The pharmacological treatments were comparable between the two groups, and this therapeutic consistency is attributable to similar clinical outcomes. Therefore, the restricted myocardial salvage beyond 6 h of PPCI was insufficient to translate into clinical outcome benefits over 5 years of follow-up. However, PPCI beyond 6 h may still relieve residual ischaemia and reduce angina burden, potentially improving quality of life despite limited long-term mortality benefit.

Findings from the present study reaffirm the critical importance of minimising total ischaemic time and underscore the necessity of timely reperfusion pathways. Future investigations should aim to validate these observations through large-scale, multicentre prospective studies, further facilitating more tailored management approaches for STEMI patients, ultimately improving both short- and long-term cardiovascular outcomes.

## Limitation

This study has several limitations. First, its retrospective, observational, single-centre design limits causal inference. Although propensity score matching was applied to reduce selection bias and improve comparability between groups, residual confounding, including confounding by indication and unmeasured variables, cannot be fully excluded. Second, symptom onset time may be imprecise in retrospective data, and some clinically relevant time intervals were not consistently available, which may limit the interpretation of timing-related findings. Third, the long study period may have introduced heterogeneity in clinical practice, treatment strategies and adjunctive therapies over time. Finally, 8.29% of patients were lost to follow-up, which may have introduced attrition bias. Future prospective, multicentre studies with larger cohorts, standardised protocols, and more complete timing and outcome data are warranted to validate these findings.

## Conclusion

PPCI performed beyond 6 h post-symptom onset is associated with improved in-hospital outcome, but not with long-term prognostic benefits. Efforts should be made to ensure PPCI as early as possible after symptom onset.

## CRediT authorship contribution statement

**Jing Gao:** Validation, Supervision. **Lin Pi:** Validation, Supervision. **Zhenxing Fan:** Validation, Investigation. **Zhi Liu:** Validation, Supervision. **Ming Yi:** Methodology, Investigation, Formal analysis. **Keling Xiao:** Investigation, Data curation. **Yinghua Zhang:** Investigation, Data curation. **Lihui Zhou:** Writing – original draft, Formal analysis, Data curation, Conceptualization. **Jing Li:** Writing – review & editing, Supervision, Funding acquisition, Conceptualization. **Jin Si:** Writing – original draft, Methodology, Formal analysis, Conceptualization. **Haoyu Zhang:** Investigation, Data curation. **Jinghao Sun:** Investigation, Data curation. **Lijie Sun:** Investigation, Data curation.

## Ethics approval and consent to participate

This study was conducted following the Declaration of Helsinki and was approved by the Ethics Committee of Xuanwu Hospital Capital Medical University (Clinical trial [2022] No.169). Given the retrospective nature of this study, the need for written informed consent was waived.

## Funding

The study was supported by grants from the 10.13039/501100001809National Natural Science Foundation of China (82170347), the Beijing Municipal Health Commission (PWD&RPP-MRI, JYY2023-13), and the Beijing Municipal Science & Technology Commission (20230484418) to Dr. Li.

## Declaration of competing interest

The authors declare the following financial interests/personal relationships which may be considered as potential competing interests: Jing Li reports financial support was provided by National Natural Science Foundation of China. Jing Li reports financial support was provided by Beijing Municipal Health Commission. Jing Li reports financial support was provided by Beijing Municipal Science and Technology Commission. If there are other authors, they declare that they have no known competing financial interests or personal relationships that could have appeared to influence the work reported in this paper.

## Data Availability

The data that support the findings of this study are not publicly available. De-identified data may be made available from the corresponding author upon reasonable request and with appropriate institutional approval. The corresponding author has full access to all the data in the study and takes responsibility for their integrity and data analysis.

## Footnotes

Appendix A Supplementary data associated with this article can be found in the online version at doi:10.1016/j.clinme.2026.100602.
