# Supplementary material for: The impact of primary percutaneous coronary intervention timing on prognosis in STEMI patients
Source: Clin Med (Lond). 2026 Jun 5;26(4):100602. doi: 10.1016/j.clinme.2026.100602 (PMC13315194; doi:10.1016/j.clinme.2026.100602)
Supplement: Supplementary file 1 — Supplementary material [file mmc1.docx]

**Supplemental Material**

**Content of Supplemental Material**

**The impact of primary percutaneous coronary intervention timing on prognosis in patients with STEMI**

**Supplemental Tables**

- Table S1 Baseline characteristics between the lost-to-follow-up cohort and the retained cohort
- Table S2 Baseline characteristics in the propensity score–matched cohort: PPCI ≤6 h vs >6 h
- Table S3 Baseline characteristics in the propensity score–matched cohort: PPCI >6 h vs no PPCI
- Table S4 Covariate balance before and after propensity score matching: PPCI ≤6 h vs >6 h
- Table S5 Covariate balance before and after propensity score matching: PPCI >6 h vs no PPCI

**Table S1. Baseline characteristics between the lost-to-follow-up cohort and the retained cohort**

| Variables | Lost-to-follow-up  (n = 295) | Retained  (n = 3266) | P value |
| --- | --- | --- | --- |
| Demographics |  |  |  |
| Age, year | 60.51 ± 12.30 | 62.31 ± 12.58 | 0.382 |
| Male, n (%) | 246 (83.4) | 2533 (77.6) | 0.020 |
| Calendar year of index hospitalization | 2014 (2010, 2017) | 2017 (2014, 2020) | < 0.001 |
| Cardiovascular risk factors |  |  |  |
| Hypertension, n (%) | 141 (47.8) | 1872 (57.3) | 0.002 |
| Diabetes mellitus, n (%) | 80 (27.1) | 1008 (30.9) | 0.181 |
| Current smoking, n (%) | 196 (66.4) | 1993 (61.0) | 0.067 |
| Obesity, n (%) | 55 (18.6) | 705 (21.6) | 0.304 |
| Initial presentation |  |  |  |
| Anterior infarction, n (%) | 157 (53.2) | 1718 (52.6) | 0.839 |
| GRACE risk score | 145.97 ± 31.61 | 147.93 ± 35.56 | 0.240 |
| Killip class ≧ 2, n (%) | 104 (35.3) | 1291 (39.5) | 0.150 |
| Early management |  |  |  |
| Aspirin, n (%) | 292 (99.0) | 3243 (99.3) | 0.576 |
| P2Y12 inhibitor, n (%) | 291 (98.6) | 3226 (98.8) | 0.245 |
| Glycoprotein IIb/IIIa inhibitor, n (%) | 78 (26.4) | 888 (27.2) | 0.845 |
| Bivalirudin, n (%) | 6 (2.0) | 85 (2.6) | 0.626 |
| Statin, n (%) | 292 (99.0) | 3224 (98.7) | 0.707 |
| ACEI/ARB/ARNI, n (%) | 278 (94.2) | 2887 (88.4) | 0.320 |
| Beta-blocker, n (%) | 249 (84.4) | 2548 (78.0) | 0.244 |
| Coronary angiography and reperfusion |  |  |  |
| Multi-vessel disease, n (%) | 195 (66.1) | 2260 (69.2) | 0.296 |
| IRA, n (%) |  |  | 0.536 |
| LAD | 151 (51.2) | 1661 (50.9) |  |
| LCX | 41 (13.9) | 389 (11.9) |  |
| RCA | 103 (34.9) | 1216 (37.2) |  |
| Auxiliary examination |  |  |  |
| LVEF, % | 56.13 ± 10.56 | 55.71 ± 9.87 | 0.077 |
| cTnI, ng/mL | 33.10 (9.12, 50.0) | 17.80 (5.49, 43.50) | 0.043 |
| LDL-C, mmol/L | 2.79 ± 0.91 | 2.74 ± 0.88 | 0.871 |
| HbA1c, % | 6.55 ± 1.60 | 6.64 ± 2.14 | 0.365 |
| hs-CRP, mg/L | 5.35 (2.10, 14.07) | 5.36 (1.94, 14.95) | 0.987 |
| IL-6, pg/mL | 18.35 (8.83, 35.41) | 19.48 (9.68, 39.42) | 0.228 |

ACEI = angiotensin-converting enzyme inhibitor; AMI = acute myocardial infarction; ARB = angiotensin receptor blocker; ARNI = angiotensin receptor-neprilysin inhibitor; cTnI = cardiac troponin I; GRACE score = Global Registry of Acute Coronary Events score; HbA1c = glycosylated hemoglobin; hs-CRP = high sensitive C reactive protein; IL-6 = interleukin-6; IRA = infarct-related artery; LAD = left anterior descending; LCX = left circumflex; LDL-C = low-density lipoprotein cholesterol; LVEF = left ventricular ejection fraction; PPCI = primary percutaneous coronary artery intervention; RCA = right coronary artery.

**Table S2. Baseline characteristics in the propensity score–matched cohort: PPCI ≤6 h vs >6 h**

| Variables | PPCI within 6h  (n = 572) | PPCI beyond 6h  (n = 572) | P value |
| --- | --- | --- | --- |
| Demographics |  |  |  |
| Age, year | 62.27 ± 12.23 | 62 ± 12.15 | 0.712 |
| Male, n (%) | 449 (78.5) | 448 (78.3) | > 0.999 |
| Calendar year of index hospitalization | 2018 (2015, 2020) | 2018 (2014, 2021) | 0.912 |
| Cardiovascular risk factors |  |  |  |
| Hypertension, n (%) | 314 (54.9) | 316 (55.2) | 0.959 |
| Diabetes mellitus, n (%) | 180 (31.5) | 186 (32.5) | 0.669 |
| Current smoking, n (%) | 338 (59.1) | 339 (59.3) | > 0.999 |
| Obesity, n (%) | 109 (19.1) | 112 (19.6) | 0.927 |
| Initial presentation |  |  |  |
| Anterior infarction, n (%) | 301 (52.6) | 301 (52.6) | > 0.999 |
| GRACE risk score | 149.46 ± 33.14 | 149.09 ± 32.48 | 0.851 |
| Killip class ≧ 2, n (%) | 226 (39.5) | 221 (38.6) | 0.808 |
| Early management |  |  |  |
| Total ischemic time, h | 4.00 (3.00, 4.92) | 8.57 (7.00, 12.47) | < 0.001 |
| Aspirin, n (%) | 564 (99.6) | 560 (99.1) | 0.307 |
| P2Y12 inhibitor, n (%) | 561 (98.1) | 567 (99.1) | 0.527 |
| Glycoprotein IIb/IIIa inhibitor, n (%) | 279 (48.8) | 267 (46.7) | 0.516 |
| Bivalirudin, n (%) | 9 (1.6) | 9 (1.6) | > 0.999 |
| Statin, n (%) | 567 (99.1) | 565 (98.8) | 0.533 |
| ACEI/ARB/ARNI, n (%) | 478 (83.6) | 471 (82.3) | 0.710 |
| Beta-blocker, n (%) | 443 (77.4) | 428 (74.8) | 0.345 |
| Coronary angiography and reperfusion |  |  |  |
| Multi-vessel disease, n (%) | 386 (67.5) | 400 (69.9) | 0.391 |
| IRA, n (%) |  |  | 0.133 |
| LAD | 318 (55.6) | 326 (57.0) |  |
| LCX | 45 (7.9) | 67 (11.7) |  |
| RCA | 209 (36.5) | 179 (31.3) |  |
| Emergent stent implantation | 1.14 ± 0.58 | 1.13 ± 0.65 | 0.920 |
| Auxiliary examination |  |  |  |
| LVEF, % | 54.19 ± 9.1 | 54.34 ± 8.82 | 0.771 |
| cTnI, ng/mL | 36.5 (20, 50) | 31.3 (12.05, 50) | 0.108 |
| LDL-C, mmol/L | 2.86 ± 0.81 | 2.82 ± 0.89 | 0.411 |
| HbA1c, % | 6.6 ± 1.53 | 6.82 ± 2.67 | 0.088 |
| hs-CRP, mg/L | 3.99 (1.61, 10.02) | 6.36 (2.14, 14.34) | < 0.001 |
| IL-6, pg/mL | 16.94 (9.61, 30.78) | 21.63 (10.46, 38.02) | 0.004 |

ACEI = angiotensin-converting enzyme inhibitor; AMI = acute myocardial infarction; ARB = angiotensin receptor blocker; ARNI = angiotensin receptor-neprilysin inhibitor; cTnI = cardiac troponin I; GRACE score = Global Registry of Acute Coronary Events score; HbA1c = glycosylated hemoglobin; hs-CRP = high sensitive C reactive protein; IL-6 = interleukin-6; IRA = infarct-related artery; LAD = left anterior descending; LCX = left circumflex; LDL-C = low-density lipoprotein cholesterol; LVEF = left ventricular ejection fraction; PPCI = primary percutaneous coronary artery intervention; RCA = right coronary artery.

**Table S3. Baseline characteristics in the propensity score–matched cohort: PPCI >6 h vs no PPCI**

| Variables | PPCI beyond 6h  (n = 565) | No PPCI  (n = 565) | P value |
| --- | --- | --- | --- |
| Demographics |  |  |  |
| Age, year | 62.39 ± 11.94 | 62.72 ± 13.19 | 0.660 |
| Male, n (%) | 435 (77) | 451 (79.8) | 0.281 |
| Calendar year of index hospitalization | 2018 (2014, 2020) | 2017 (2015, 2020) | 0.573 |
| Cardiovascular risk factors |  |  |  |
| Hypertension, n (%) | 319 (56.5) | 306 (54.2) | 0.460 |
| Diabetes mellitus, n (%) | 193 (34.2) | 185 (32.7) | 0.661 |
| Current smoking, n (%) | 334 (59.1) | 327 (57.9) | 0.710 |
| Obesity, n (%) | 111 (19.6) | 114 (20.2) | 0.795 |
| Initial presentation |  |  |  |
| Anterior infarction, n (%) | 298 (52.7) | 312 (55.2) | 0.435 |
| GRACE risk score | 149.78 ± 32.61 | 150.27 ± 36.91 | 0.814 |
| Killip class ≧ 2, n (%) | 223 (39.5) | 224 (39.6) | > 0.999 |
| Early management |  |  |  |
| Aspirin, n (%) | 560 (99.1) | 560 (99.1) | > 0.999 |
| P2Y12 inhibitor, n (%) | 560 (99.1) | 559 (98.9) | 0.270 |
| Glycoprotein IIb/IIIa inhibitor, n (%) | 263 (46.5) | 54 (9.6) | < 0.001 |
| Bivalirudin, n (%) | 8 (1.4) | 15 (2.7) | 0.195 |
| Statin, n (%) | 557 (98.6) | 557 (98.6) | > 0.999 |
| ACEI/ARB/ARNI, n (%) | 465 (82.3) | 488 (86.4) | 0.184 |
| Beta-blocker, n (%) | 423 (74.9) | 437 (77.3) | 0.363 |
| Coronary angiography and reperfusion |  |  |  |
| Multi-vessel disease, n (%) | 404 (71.5) | 405 (71.7) | > 0.999 |
| IRA, n (%) |  |  | 0.005 |
| LAD | 314 (55.6) | 352 (62.3) |  |
| LCX | 44 (7.8) | 57 (10.1) |  |
| RCA | 207 (36.6) | 156 (27.6) |  |
| Auxiliary examination |  |  |  |
| LVEF, % | 54.43 ± 8.91 | 54.57 ± 10.2 | 0.816 |
| cTnI, ng/mL | 31.7 (12.15, 50) | 7.7 (2.42, 17.58) | < 0.001 |
| LDL-C, mmol/L | 2.81 ± 0.89 | 2.63 ± 0.86 | 0.009 |
| HbA1c, % | 6.86 ± 2.69 | 6.63 ± 1.62 | 0.076 |
| hs-CRP, mg/L | 6.63 (2.17, 15.47) | 8.38 (2.22, 27.43) | 0.022 |
| IL-6, pg/mL | 21.89 (10.54, 39.7) | 24.76 (10.29, 49.44) | 0.212 |

ACEI = angiotensin-converting enzyme inhibitor; AMI = acute myocardial infarction; ARB = angiotensin receptor blocker; ARNI = angiotensin receptor-neprilysin inhibitor; cTnI = cardiac troponin I; GRACE score = Global Registry of Acute Coronary Events score; HbA1c = glycosylated hemoglobin; hs-CRP = high sensitive C reactive protein; IL-6 = interleukin-6; IRA = infarct-related artery; LAD = left anterior descending; LCX = left circumflex; LDL-C = low-density lipoprotein cholesterol; LVEF = left ventricular ejection fraction; PPCI = primary percutaneous coronary artery intervention; RCA = right coronary artery.

**Table S4. Covariate balance before and after propensity score matching: PPCI ≤6 h vs >6 h**

| Covariate | SMD before matching | SMD after matching |
| --- | --- | --- |
| Propensity score distance | 0.355 | 0.030 |
| Age | -0.124 | 0.022 |
| Sex | -0.043 | -0.002 |
| Calendar year of index hospitalization | -0.195 | -0.010 |
| Hypertension | -0.057 | -0.005 |
| Diabetes mellitus | -0.193 | -0.026 |
| Current smoking | 0.039 | -0.002 |
| Obesity | 0.105 | 0.004 |
| Killip class ≧ 2 | -0.014 | 0.009 |
| Multivessel disease | -0.146 | -0.054 |
| GRACE score | -0.126 | 0.012 |
| LVEF | 0.115 | -0.017 |
| IL-6 | -0.031 | 0.025 |

GRACE score = Global Registry of Acute Coronary Events score; IL-6 = interleukin-6; LVEF = left ventricular ejection fraction; PPCI = primary percutaneous coronary intervention; SMD = standardized mean difference.

**Table S5. Covariate balance before and after propensity score matching: PPCI >6 h vs no PPCI**

| Covariate | SMD before matching | SMD after matching |
| --- | --- | --- |
| Propensity score distance | 0.442 | 0.001 |
| Age | -0.141 | -0.027 |
| Sex | -0.023 | 0.028 |
| Calendar year of index hospitalization | 0.335 | -0.002 |
| Hypertension | -0.099 | 0.045 |
| Diabetes mellitus | 0.013 | 0.029 |
| Current smoking | -0.020 | 0.012 |
| Obesity | -0.053 | -0.021 |
| Killip class ≧ 2 | -0.047 | -0.002 |
| Multivessel disease | -0.056 | -0.011 |
| GRACE score | -0.050 | -0.015 |
| LVEF | -0.224 | -0.015 |
| IL-6 | -0.124 | 0.005 |

GRACE score = Global Registry of Acute Coronary Events score; IL-6 = interleukin-6; LVEF = left ventricular ejection fraction; PPCI = primary percutaneous coronary intervention; SMD = standardized mean difference.
